# MiR-23a regulates TGF-β-induced epithelial-mesenchymal transition by targeting E-cadherin in lung cancer cells

**DOI:** 10.3892/ijo.2012.1535

**Published:** 2012-06-28

**Authors:** MENGRU CAO, MASAHIRO SEIKE, CHIE SOENO, HIDEAKI MIZUTANI, KAZUHIRO KITAMURA, YUJI MINEGISHI, RINTARO NORO, AKINOBU YOSHIMURA, LI CAI, AKIHIKO GEMMA

**Affiliations:** 1 Department of Internal Medicine, Division of Pulmonary Medicine/Infection and Oncology, Nippon Medical School, Tokyo 113-8603, Japan;; 2 Department of Medical Oncology, The Third Affiliated Hospital of Harbin Medical University, Harbin, P.R. China

**Keywords:** microRNA, epithelial-mesenchymal transition, transforming growth factor-β, Smad, lung cancer

## Abstract

Transforming growth factor-β (TGF-β)-induced epithelial-mesenchymal transition (EMT) has been shown to be related to the pathogenesis of various diseases including lung cancer. Recently, microRNAs (miRNA) have been recognized as a new class of genes involved in human tumorigenesis. MiR-23a/24/27a is a miRNA cluster located in chromosome 19p13.12, which can function as an oncogene in several human cancers. In this study, we analyzed miR-23a/24/27a expression in 10 non-small cell cancer (NSCLC) cell lines by real-time PCR analysis. Correlation between expression of these miRNAs and TGF-β/Smad signaling was evaluated. We found that miR-23a could be regulated by TGF-β1 in a Smad-dependent manner in A549 lung adenocarcinoma cells showing the EMT phenomenon. Knockdown of miR-23a partially restored E-cadherin expression under conditions of TGF-β1 stimulation. In contrast, overexpression of miR-23a could suppress E-cadherin expression and stimulate EMT. Furthermore, A549 cells with overexpressed miR-23a were more resistant to gefitinib compared to the parental cells. These findings suggest that miR-23a regulates TGF-β-induced EMT by targeting E-cadherin in lung cancer cells and may be useful as a new therapeutic target in NSCLC.

## Introduction

Lung cancer continues to be a leading cause of cancer death both in Japan and worldwide (1) and, despite recent improvements in chemotherapies and molecular-targeted therapies, the prognosis remains poor (2–5). Patient selection based on a specific biomarker is one strategy that could lead to improved lung cancer treatments. Although some biomarkers predictive of metastasis, prognosis and drug sensitivity have already been reported in lung cancer, more sensitive and specific biomarkers could facilitate the development of novel therapeutic applications (6–8).

Epithelial-mesenchymal transition (EMT) comprises a complex series of reversible events that can lead to the loss of epithelial cell adhesion and the induction of a mesenchymal phenotype (9). Thus, EMT is characterized by the loss of epithelial differentiation markers including E-cadherin and the induction of mesenchymal markers such as vimentin and fibronectin. EMT can be induced by transforming growth factor-β1 (TGF-β1) (10). The Smad pathway is a major transducer of TGF-β signaling (11). Smad2 and Smad3 are phosphorylated by the TGF-β type I receptor and form complexes with Smad4 (11). These complexes accumulate in the nucleus of the cell, regulating the transcription of target genes and playing critical roles in the control of cell proliferation, differentiation, apoptosis and cell migration. In response to TGF-β, the TGF-β receptors also activate alternative signaling effectors, such as mitogen-activated protein kinase, phosphatidylinositol-3 kinase, and Rho-like GTPases (11). It has been recognized that EMT plays a pivotal role in several diverse processes during embryonic development, chronic inflammation and fibrosis (12). Recently, several studies demonstrated that EMT was correlated with carcinogenesis, metastasis and poor prognosis in various human cancers, including those of the lung (13–16). Furthermore, EMT has been reported to be related to reduced sensitivity and acquired resistance to epidermal growth factor tyrosine kinase inhibitors (EGFR-TKI) in lung cancer cells (17–19). Taken together, these findings demonstrate that the suppression of EMT could be used as a potential target for treatment of lung cancer.

MicroRNA (miRNAs) are a class of short single-stranded noncoding endogenous RNAs, approximately 18–24 nucleotides in length, which post-transcriptionally modulate gene expression by either inhibiting translation or inducing mRNA degradation (20). MiRNAs have been recognized as a new class of genes involved in human tumorigenesis (21,22) and recently they have been shown to be diagnostic, prognostic and therapeutic biomarkers in lung cancer (22–25). For example, high miR-155 expression and low let-7a expression, as independent risk factors, have a negative prognostic impact on outcome in lung adenocarcinoma patients (23). The miR-17-92 cluster functions as an oncogene, and has been shown to promote lung cancer carcinogenesis (24). We previously reported that the inhibition of miR-21, whose upregulation is associated with EGFR mutations, can be a therapeutic strategy, either as a monotherapy or in combination with EGFR-TKI treatment (25). These findings suggest that miRNA can serve as a novel therapeutic target as well as diagnostic and prognostic marker in lung cancer.

A recent study reported that a specific cluster of miRNA, miR-23a/24/27a, was induced by TGF-β in a Smad-dependent manner in hepatocellular carcinoma (HCC) cells (26). Upregulation of these miRNAs were able to suppress TGF-β-induced growth suppressive activities in HCC cells. In this present study, we analyzed miR-23a/24/27a expression in non-small cell cancer (NSCLC) cells and evaluated the correlation between its expression and TGF-β/Smad signaling. We found that miR-23a could be induced by TGF-β in a Smad-dependent manner in A549 cells. In addition, overexpression of mature miR-23a reduced E-cadherin expression and stimulate the EMT phenomenon which is involved in tumorigenesis. Furthermore, in these A549 cells, inhibition of miR-23a could also partially suppress TGF-β-induced EMT, while overexpression was associated with the tendency for EGFR-TKI resistance. We have demonstrated that, in lung cancer cells, miR-23a is regulated by the TGF-β/Smad pathway and plays a critical role in EMT through the targeting of E-cadherin.

## Materials and methods

### Cell culture

We used 10 NSCLC cell lines: A549, PC9, PC14, LC2/ad, RERF-LC-KJ, RERF-LC-OK adenocarcinoma (AC) cell lines and PC1, PC10, LK2 and SQ5 squamous cell carcinoma cell lines (SCC) for this study. An immortalized tracheal cell line (BET2A) was used as a normal control cells. A549 and BET2A were purchased from the American Type Culture Collection (ATCC, Manassas, VA, USA); RERF-LC-KJ, RERF-LC-OK, LC2-ad, LC2/Ad and SQ5 were obtained from the RIKEN Cell Bank (Ibaraki, Japan); and PC1, PC9, PC10 and PC14 were obtained from Immuno-Biological laboratories (Gunma, Japan). NSCLC cell lines were maintained in RPMI-1640 medium (Gibco, Carlsbad, CA, USA) supplemented with 10% fetal bovine serum (FBS). BET2A was maintained in RPMI-1640 medium with 5% FBS.

### RNA extraction and real-time quantitative reverse transcription-PCR

Total RNA was extracted from the BET2A and NSCLC cell lines with Trizol reagent (Invitrogen, Carlsbad, CA, USA). The miR-23a, miR-24 and miR-27a expression levels were quantified by quantitative reverse transcription-PCR (qRT-PCR) using TaqMan^®^ MicroRNA Assay System (Applied Biosystems, Foster City, CA). RNU66 (PN 4373382) was used as an internal control (Applied Biosystems). MiRNA expression was quantified and reported as 2^−ΔΔCt^ value (27).

### Antibodies and western blot analysis

Cells were lysed in buffer containing 50 mM Tris-HCl, pH 7.6, 150 mM NaCl, 0.1% sodium dodecyl sulfate, 1% Nonidet P-40, and 0.5% sodium-deoxycholate. The lysates were kept on ice for 30 min, and then centrifuged at 13000 × g for 30 min. The supernatant was collected and then 10 *μ*g of each of the proteins was separated by SDS-PAGE on 10% gels and transferred to nitrocellulose membrane. After being blocked in 5% skimmed milk, the membrane was incubated with Smad2/3, β-actin (Cell Signaling Technology, Beverley, MA, USA), E-cadherin, N-cadherin and vimentin (Santa Cruz Biotechnology, Santa Cruz, CA, USA) antibodies. Proteins were detected by immunoblotting using ECL-Plus reagents (GE Healthcare Bio-Science Corp, Piscataway, NJ, USA).

### Oligonucleotide transfection

TGF-β1 was purchased from R&D system (Minneapolis, MN, USA). Cells were treated with 5 ng/ml TGF-β1 for the indicated time. Small interference RNA (siRNA) targeting Smad2/3 was purchased from Dharmacon Research Inc. (Lafayette, CO, USA) and the homologous negative control was obtained from Invitrogen. MiR-23a inhibitor, its negative control, miR-23a precursor (Pre-miR-23a) and its cognate negative control (Pre-miR-ctl) were synthesized by Ambion (Ambion). Pre-miR-23a and miR-23a inhibitor were transfected using Lipofectamine™ 2000 reagent 24 h after seeding, as per the manufacturer’s instructions (Invitrogen). Transfections of precursor and inhibitor complexes were added to cells at a final concentration of 40 nM. Six hours after the transfection was performed, the transfection medium was replaced, and after 24 h, 5 ng/ml TGF-β1 was added to the medium which was then incubated at 37°C for 48 h.

### Growth inhibition assay

Gefitinib was purchased from Seleck Chemicals (Houston, TX, USA). A549 cells (5,000 cells/well) were seeded into 96-well plates for 24 h. After being treated with Pre-miR-ctl or Pre-miR-23a, at a final concentration of 40 nM for 24 h, the cells were incubated in the various concentrations of gefitinib for 72 h at 37°C. Then, MTS was added to each well and the cells were incubated for a further 2 h at 37°C, after which absorbance was measured using a microplate reader with a test wavelength of 450 nm. The IC_50_ value was defined as the concentration needed for 50% reduction of the growth by treatment with gefitinib.

## Results

### MiR-23a, miR-24 and miR-27a expression in lung cancer cells

**Figure 1:**
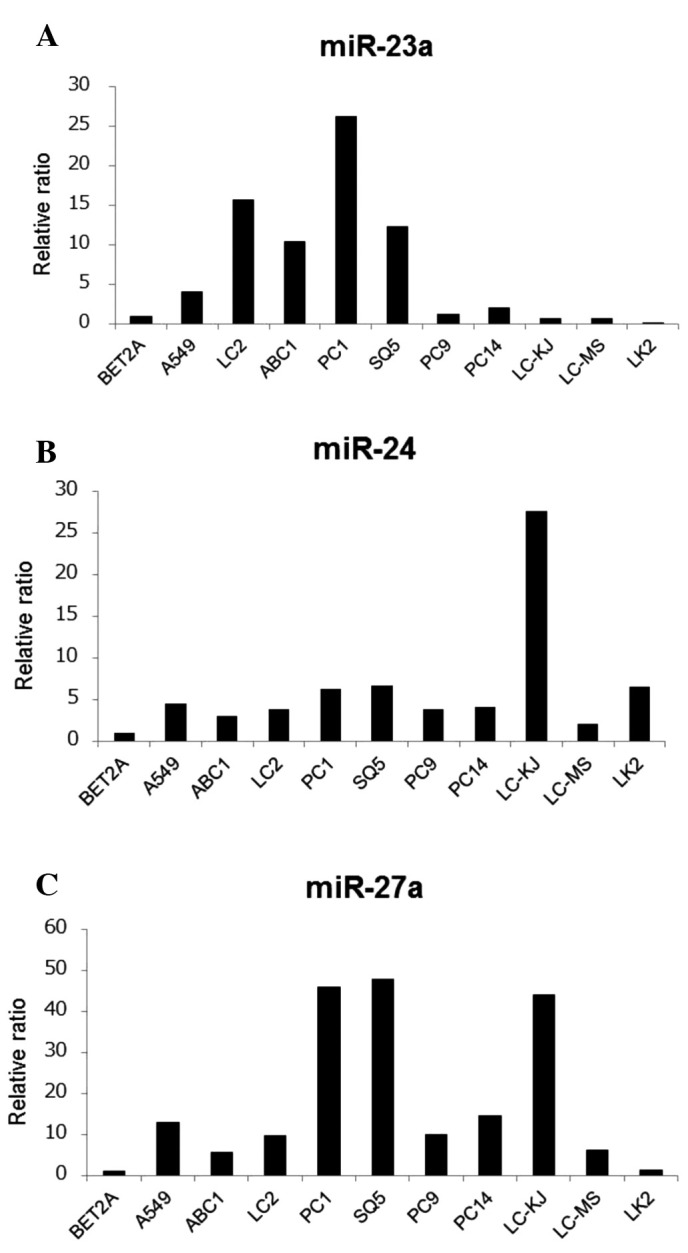
MiR-23a/24/27a expression in NSCLC cells. Expression levels of miR-23a/24/27a were analyzed in 10 NSCLC cells and BET2A cells by TaqMan^®^ MicroRNA assay. MiRNA expression was quantified as 2^−ΔΔCt^ value. Relative expressions of miR-23a/24/27 against BET2A cells are shown.

We first investigated miR-23a, miR-24 and miR-27a expression levels in NSCLC cell lines, including 6 AC cell lines and 4 SCC cell lines. Expression levels of these mature miRNAs were examined by qRT-PCR (Fig. 1). Although these miRNAs belonged to the same miRNA cluster, their expression levels varied among the 10 cell lines.

### MiR-23a expression is directly induced by TGF-β1 in a Smad-dependent manner

**Figure 2:**
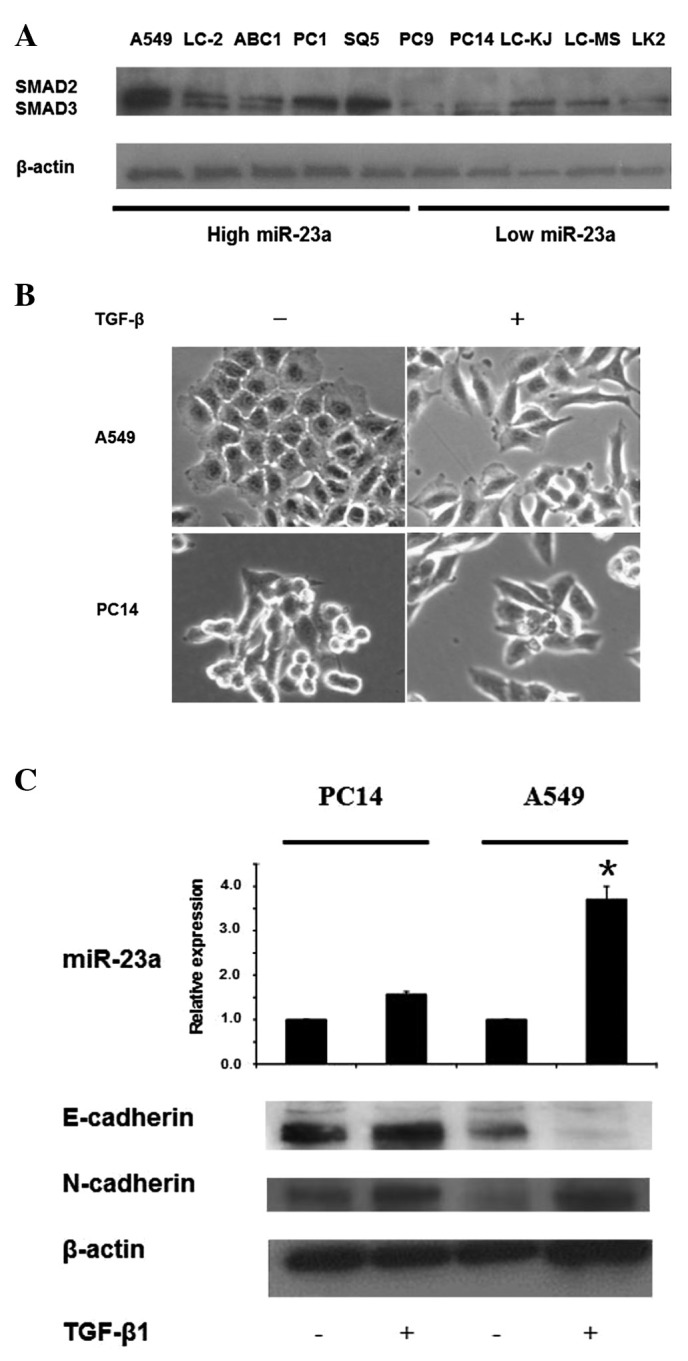

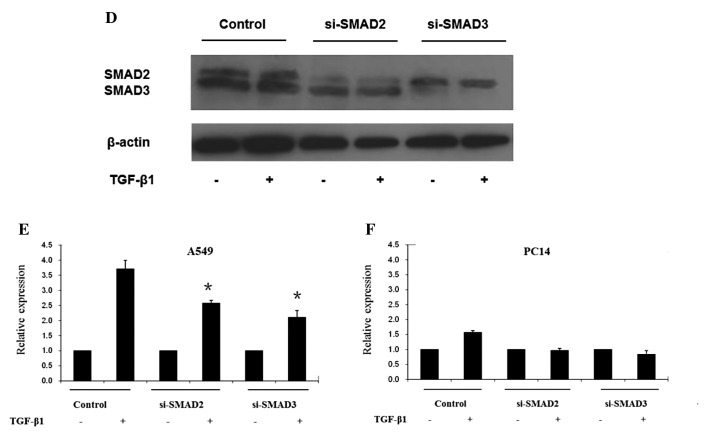
MiR-23a is directly induced by TGF-β1 in a SMAD-dependent manner and involved in EMT of lung cancer cells. (A) Five cell lines (A549, LC-2, ABC1, PC1 and SQ5) showed high expression of both miR-23a and Smad2/3. In contrast, low expression of miR-23a and Smad2/3 were found in the other five cell lines (PC9, PC14, LC-KJ, LC-MS and LK2). (B) A549 and PC14 cells were treated with 5 ng/ml of TGF-β1 for 48 h. The cells were observed under a light microscope. A549 cells exhibited a classic epithelial morphology. In contrast, A549 cells after the TGF-β1 exposure appeared to be less uniformly epithelial. (C) MiR-23a and protein expression of EMT markers in A549 and PC14 cells after TGF-β1 stimulation. Compared with PC14 cells, high expression of miR-23a, lost E-cadherin expression and enhanced N-cadherin expression were observed in A549 cells after TGF-β1 stimulation. (D) Protein expression of Smad2/3 after treatment of siRNA in A549 cells. A549 cells were treated with control siRNA or siRNA of Smad2/3 with or without TGF-β1 stimulation. Smad2 or Smad3 specific siRNA completely diminished Smad2 or Smad3 expression. (E) Expression of miR-23a after the transfection of Smad2/3 siRNA was evaluated. MiR-23a expression was significantly decreased after the treatment of Smad2 or Smad3 siRNAs with TGF-β1 stimulation in A549 cells. (F) MiR-23a expression was unchanged after the transfection of Smad2 or Smad3 siRNAs with TGF-β1 stimulation in PC14 cells. Data are mean ± SD from 3 independent experiments. ^*^p<0.05 when compared with the respective parent cells.

A recent study reported that miR-23a/24/27a was induced by TGF-β in a Smad-dependent manner in HCC cells (26). Smad pathway is known as a major transducer of TGF-β signaling (11). We evaluated the correlation between miR-23a, miR-24 and miR-27a expression levels and Smad expression. High expression of miR-23a was observed in those NSCLC cells which also overexpressed Smad2/3. Thus, 5 cell lines (A549, LC-2/ad, ABC1, PC1 and SQ5) showed high expression of miR-23a and Smad2/3 (Fig. 2A), while low expression of miR-23a and Smad2/3 was found in the other 5 cell lines (PC9, PC14, LC-KJ, LC-MS and LK2) (Fig. 2A). In contrast, no correlation was observed between miR-24 or miR-27a and Smad2/3.

It is well known that TGF-β1 stimulates the EMT of A549 lung cancer cells (10). In this study, A549 cells which over-expressed miR-23a and Smad2/3 was treated with 5 ng/ml of TGF-β1 for 48 h. We observed that, while the parent A549 cells exhibited a classic epithelial morphology (Fig. 2B), after TGF-β1 exposure they had a less uniform epithelial appearance (Fig. 2B). In contrast, PC14 cells with low expression of miR-23a and Smad2/3 retained their epithelial morphology after TGF-β1 treatment (Fig. 2B). Using western blot analysis, we evaluated the expression levels of EMT markers in A549 and PC14 cells treated with TGF-β1 in order to confirm the occurrence of EMT. A549 cells treated with TGF-β1 displayed reduced E-cadherin expression and increased N-cadherin expression when compared with A549 cells without TGF-β1 stimulation (Fig. 2C). We also examined whether TGF-β1 stimulated miR-23a expression in these two cells. MiR-23a expression level was significantly higher in A549 cells, which had shown the EMT phenomenon after the treatment of the respective parent cells with TGF-β1 (Fig. 2C). In contrast, miR-23a expression level in PC14 cells, which had not shown the EMT phenomenon, was unaffected by exposure to TGF-β1 (Fig. 2C).

Next, we examined whether the Smad signal pathway directly regulated miR-23a expression. A549 and PC14 cells were treated with siRNAs of control or Smad2/3 (Fig. 2D). Knockdown of Smad2/3 significantly decreased TGF-β1-induced miR-23a expression in A549 cells in which miR-23a was overexpressed (Fig. 2E). On the other hand, in PC14 cells with low miR-23a, miR23a expression was unaffected by treatment with Smad siRNAs (Fig. 2F). These results suggested that, in A549 lung cancer cells, miR-23a was directly regulated by TGF-β1/Smad pathway and contributed to the EMT phenomenon.

### MiR-23a regulates TGF-β1-induced EMT by targeting E-cadherin

**Figure 3:**
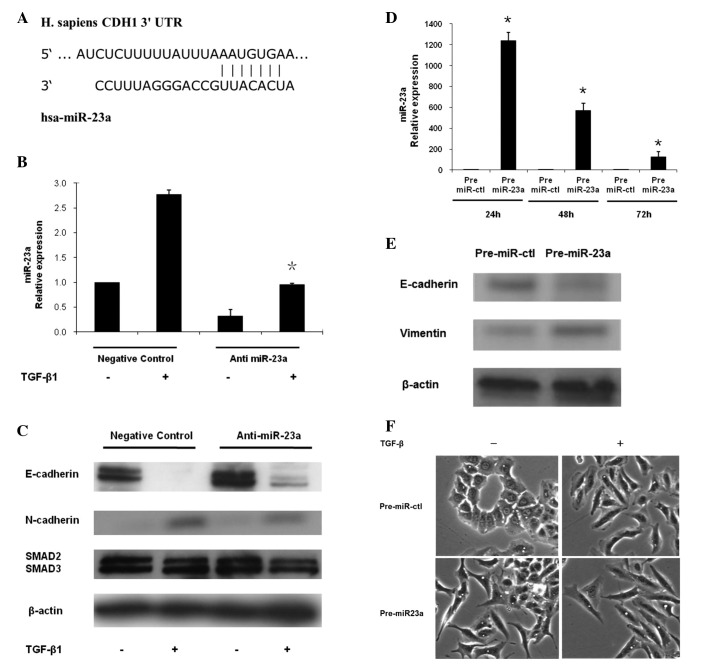
MiR-23a directly targets CDH1 and regulated TGF-β1-induced EMT. (A) Predicted duplex formation between human CDH1 3′ UTR and miR-23a. (B) Control or specific miR-23a inhibitor was transfected into A549 cells for 24 h, which were then treated with or without TGF-β1 for a further 48 h. MiR-23a was significantly knocked down by miR-23a inhibitor in A549 cells. (C) EMT markers and SMAD2/3 expression were examined in A549 cells after the treatment with anti-miR-23a or control inhibitor. (D) Pre-miR-ctl or Pre-miR-23a was transfected into A549 cells for 72 h. The histograms show the relative expression of mature miR-23a in A549 cells after treatment with Pre-miR-23a or Pre-miR-ctl, by qRT-PCR analysis. (E) At 48 h, E-cadherin expression was reduced in A549 cells treated with Pre-miR-23a. In contrast, at 48 h, vimentin expression was increased in A549 cells transfected with Pre-miR-23a. (F) The cells were observed under a light microscope. A549 cells after the treatment of Pre-miR-23a appeared to have a spindle morphology.

Since miR-23a was significantly upregulated in A549 cells after the treatment with TGF-β1 and mediated EMT, we proceeded to identify potential targets known to play a role in EMT by using the Target Scan database. Among the candidate miRNAs for the E-cadherin gene (CDH1), we found that the region of 3′ UTR of the CDH1 gene may serve as a binding site for miR-23a based on the prediction of Target Scan database (Fig. 3A).

To examine whether the CDH1 was a target of miR-23a, we knocked down miR-23a in A549 cells by using a specific inhibitor. Control or specific miR-23a inhibitor was transfected into A549 cells for 24 h, which were then treated with or without TGF-β1 for a further 48 h. We confirmed that miR-23a was effectively knocked down by miR-23a inhibitor in A549 cells (Fig. 3B). Using western blot analysis, we evaluated the expression of EMT markers after the treatment of miR-23a inhibitor to confirm the occurrence of EMT. After exposure to TGF-β1, E-cadherin expression in A549 cells was greatly diminished, resulting in TGF-β1-induced EMT (Fig. 3C). Interestingly, E-cadherin was still expressed in A549 cells transfected with miR-23a inhibitor after TGF-β1 exposure (Fig. 3C). N-cadherin expression was also weak after miR-23a treatment followed by TGF-β1 exposure (Fig. 3C). These findings demonstrated that miR-23a inhibition partially suppressed TGF-β-induced EMT phenomenon in A549 cells.

We transiently transfected A549 cells with the miR-23a precursor (Pre-miR-23a), and the control precursor miR (Pre miR-ctl). Mature miR-23a was remarkably induced by the miR-23a precursor in A549 cells between 24 to 72 h (Fig. 3D). After the treatment of precursor miR-23a, decreased E-cadherin expression and increased levels of vimentin were observed in A549 cells at 48 h (Fig. 3E). Under a light microscope, overexpression of miR-23a enhanced the spindle integration, resulting in an additive effect with TGF-β1-induced EMT in A549 cells (Fig. 3F). These results suggested that miR-23a may affect EMT by targeting E-cadherin in lung cancer cells.

### MiR-23a stimulated EMT and induced resistance to gefitinib

**Figure 4:**
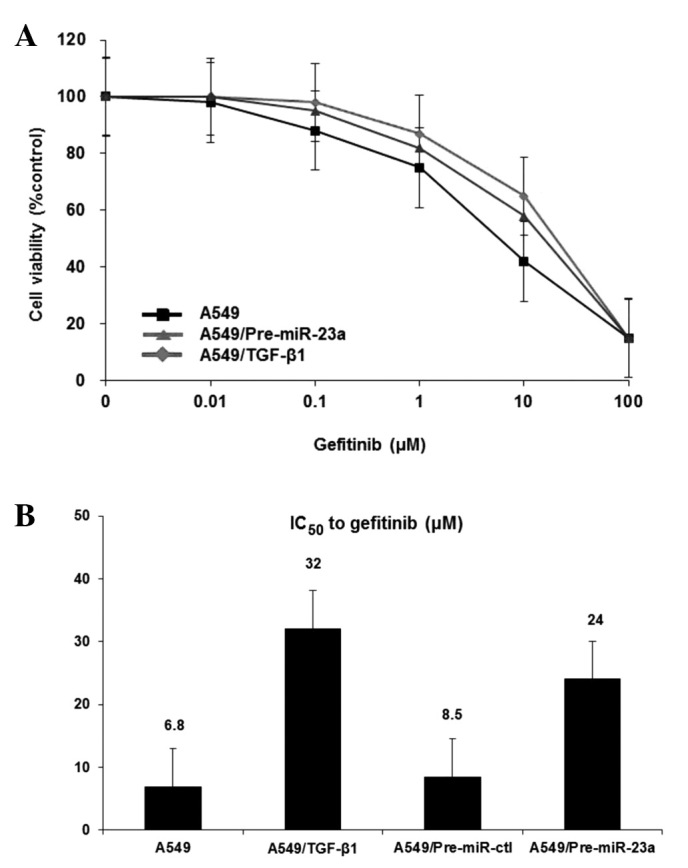
MiR-23a contributes to gefitinib drug resistance. (A) Gefitinib treatment with 5 ng TGF-β1 or Pre-miR-23a or Pre-miR-ctl for 24 h was examined in A549 cells. Then, the cells were incubated in the various concentrations of gefitinib for 72 h. (B) The IC_50_ values of gefitinib with TGF-β1 and gefitinib after treatment with Pre-miR-ctl or Pre-miR-23a. Each result is expressed as cell viability in the treated samples compared with the untreated sample (100%) for gefitinib therapy. Data are mean ± SD from 3 independent experiments.

Finally, to evaluate whether EMT leads to resistance against the EGFR-TKI, gefitinib, we measured the response to gefitinib after the exposure of A549 cells to TGF-β1 or Pre-miR-23a. Consistent with previous studies, A549 cells treated with TGF-β1 were more resistant to gefitinib than were the parental cells (Fig. 4A). Interestingly, the resistance to gefitinib was also found in A549 cells after treatment with Pre-miR-23a only (Fig. 4A). The IC_50_ values of gefitinib with TGF-β1 and gefitinib after treatment with Pre-miR-23a were 32 and 24, respectively, whereas that of gefitinib monotherapy was 6.8 (Fig. 4B). These findings indicate that induction of EMT affects cellular response to gefitinib and that miR-23a contributes to the resistance as well as TGF-β1.

## Discussion

TGF-β1 has been recognized as a regulator of EMT in advanced-stage human cancers, a phenomenon which promotes tumorigenesis, cancer progression and metastasis (10). Previous studies have demonstrated that EMT plays a key role in lung cancer progression (15,28,29). In addition, it has been demonstrated that cancer progression might stimulate EMT in lung cancer, resulting in resistance to anticancer drugs (17–19). Therefore, the early detection of EMT development or attenuation of the EMT phenotype in lung cancer cells may be useful, particularly in helping to improve the clinical treatment of lung cancer.

Recent reports showed that several miRNAs play a crucial role in the regulation of EMT of several cancers (30–34). The miR-200 family and miR-205 have been shown to contribute to EMT in cancer cells by the direct targeting of transcriptional repressors of E-cadherin, ZEB1 and ZEB2 (30–32). More specifically, in breast cancer cells, miR-155 has been shown to facilitate TGF-β-induced EMT by targeting RhoA (33). Finally, miR-9 activated by MYC/MYCN mediated E-cadherin down-regulation resulting in the activation of β-catenin, and VEGF, and metastases in human cancers, including neuroblastomas and breast tumors (34). However, in lung cancer, the mechanism by which miRNA contributes to TGF-β-induced EMT is largely unknown.

MiR-23a/24/27a is a miRNA cluster located in chromosome 19p13.12 and can be induced by TGF-β (26). This cluster functions as an oncogenic miRNA in several human cancers, and previous studies have reported that miR-23a/24/27a was upregulated in human cancers (26,35). Furthermore, miR-23a/24/27a functioned as a growth-promoting and anti-apoptotic factor in HCC cells (26), while miR-23a was also shown to promote the growth of gastric adenocarcinoma cells and downregulate interleukin-6 receptor (35). In addition, c-myc suppression of miR-23a enhances mitochondrial glutamine metabolism and glutaminase expression (36). The cognate of glutamine is the major component that catabolizes glutamine to generate energy and lactate. Plenty of large amounts of glutamine are aggressively transported into cells to promote cancer cell proliferation and act as a source of carbon in the carbon cycle. Taken together, miR-23a/24/27a can be induced by TGF-β and act as an oncogenic or tumor repressive miRNA in multiple human malignancies. However, the relation between miR-23a/24/27a and TGF-β/Smad pathway remains unclear in lung cancer cells.

In this study, we found that expression of miR-23a was directly induced by the TGF-β1/Smad pathway in A549 lung adenocarcinoma cells with the EMT phenomenon. In contrast, miR-24 and miR-27a belonging to the same cluster were induced in a Smad-independent manner in lung adenocarcinoma cells. We suggest that TGF-β1 mainly regulates the expression of miR-23a in lung cancer cells. Furthermore, overexpression of miR-23a decreased E-cadherin expression and increased levels of vimentin, resulting in the EMT phenomenon in A549 lung cancer cells; silencing of miR-23a partially restored E-cadherin expression. This is the first report showing that miR-23a regulated TGF-β1-induced EMT via E-cadherin suppression in lung cancer cells.

Molecular-targeted therapies have been recently developed for NSCLC treatment. NSCLC patients with EGFR gene mutations have shown a dramatic response to EGFR-TKIs such as gefitinib and erlotinib (4,5). We have recently reported that first-line gefitinib for advanced NSCLC patients with EGFR mutations improved progression-free survival with acceptable toxicity (5,37). However, it is recognized that, clinically, drug resistance eventually emerges and this limits the mean duration of response. Although mechanisms of acquired resistance, such as T790M secondary mutation and MET amplification, have recently been found, other mechanisms should be identified to widen the therapeutic strategy for NSCLC with EGFR mutations (38,39). EMT has been reported to be correlated with an unfavorable prognosis for NSCLC patients (40). Some studies showed that the mesenchymal phenotype was more resistant to EGFR-TKI than the epithelial phenotype in NSCLC (17–19). Similarly, restoration of E-cadherin increased the sensitivity to EGFR-TKI in lung cancer cell lines (41). Consistent with previous findings, induction of EMT by TGF-β1 in A549 cells led to the acquisition of resistance to gefitinib. Furthermore, overexpression of miR-23a induced EMT by suppressing E-cadherin expression and contributed to the reduced sensitivity to gefitinib in A549 cells. These findings demonstrated that suppression of EMT by miR-23a inhibition might overcome the resistance to EGFR-TKIs observed in NSCLC.

In conclusion, our study has provided evidence that miR-23a regulated TGF-β-induced EMT by suppression of E-cadherin and contributed to EGFR-TKI resistance in lung cancer cells. MiR-23a might be a potential prognostic marker and a new therapeutic target in NSCLC. Further studies should be performed to clarify the connection between miR-23a and TGF-β/Smad signaling during the EMT process in NSCLC.
